# The serine protease HtrA1 cleaves misfolded transforming growth factor β–induced protein (TGFBIp) and induces amyloid formation

**DOI:** 10.1074/jbc.RA119.009050

**Published:** 2019-06-13

**Authors:** Ebbe Toftgaard Poulsen, Nadia Sukusu Nielsen, Carsten Scavenius, Emilie Hage Mogensen, Michael W. Risør, Kasper Runager, Marie V. Lukassen, Casper B. Rasmussen, Gunna Christiansen, Mette Richner, Henrik Vorum, Jan J. Enghild

**Affiliations:** ‡ Department of Molecular Biology and Genetics, Aarhus University, 8000 Aarhus, Denmark; § Interdisciplinary Nanoscience Center, Aarhus University, 8000 Aarhus, Denmark; ¶ Department of Biomedicine, Aarhus University, 8000 Aarhus, Denmark; ‖ Department of Ophthalmology, Aalborg University Hospital, 9000 Aalborg, Denmark

**Keywords:** cornea, protein folding, protein misfolding, protein turnover, protease, BIGH3, corneal dystrophy, HTRA1, granular corneal dystrophy (GCD), high-temperature requirement protein A1 (HtrA1), transforming growth factor β–induced protein (TGFBIp), lattice corneal dystrophy (LCD), amyloid, eye disorder, proteolytic processing

## Abstract

The serine protease high-temperature requirement protein A1 (HtrA1) is associated with protein-misfolding disorders such as Alzheimer's disease and transforming growth factor β–induced protein (TGFBIp)–linked corneal dystrophy. In this study, using several biochemical and biophysical approaches, including recombinant protein expression, LC-MS/MS and 2DE analyses, and thioflavin T (ThT) fluorescence assays for amyloid fibril detection, and FTIR assays, we investigated the role of HtrA1 both in normal TGFBIp turnover and in corneal amyloid formation. We show that HtrA1 can cleave WT TGFBIp but prefers amyloidogenic variants. Corneal TGFBIp is extensively processed in healthy people, resulting in C-terminal degradation products spanning the FAS1-4 domain of TGFBIp. We show here that HtrA1 cleaves the WT FAS1-4 domain only inefficiently, whereas the amyloidogenic FAS1-4 mutations transform this domain into a considerably better HTRA1 substrate. Moreover, HtrA1 cleavage of the mutant FAS1-4 domains generated peptides capable of forming *in vitro* amyloid aggregates. Significantly, these peptides have been previously identified in amyloid deposits *in vivo*, supporting the idea that HtrA1 is a causative agent for TGFBIp-associated amyloidosis in corneal dystrophy. In summary, our results indicate that TGFBIp is an HtrA1 substrate and that some mutations in the gene encoding TGFBIp cause aberrant HtrA1-mediated processing that results in amyloidogenesis in corneal dystrophies.

## Introduction

Transforming growth factor beta-induced protein (TGFBIp, UniProt Q15582) is one of the most abundant proteins in the human cornea (1). In addition to the full-length protein, truncated TGFBIp fragments are observed after immunoblotting, most likely as a result of proteolysis. The smallest fragment observed comprises the C-terminal domain of TGFBIp, known as the FAS1-4 domain (2). The physiological role of TGFBIp in the cornea is not understood but may involve extracellular matrix cell adhesion involving several integrin-binding motifs (3, 4) and a C-terminal RGD integrin-binding motif. In addition, TGFBIp binds covalently to type XII collagen via a reducible bond (5). Knocking out TGFBIp in mice showed little effect on corneal integrity and mouse behavior in general (6). TGFBIp is involved in the pathophysiology of the amyloid corneal disease lattice corneal dystrophy (LCD)^3^ and amorphous granular corneal dystrophy (GCD). For a review, see Ref. 7. These diseases are characterized by TGFBIp misfolding and/or aggregation and the accumulation of insoluble protein deposits leading to visual impairment. The misfolding and/or aggregation of TGFBIp can be caused by 70 different mutations (8) mainly situated in the first FAS1 domain (FAS1-1) or in the fourth FAS1 domain (FAS1-4) of TGFBIp. Several of these mutations affect the thermodynamic stability of the protein (9–11). An increase in thermodynamic stability has been linked to mutations associated with GCD that cause amorphous aggregates, whereas a decrease in thermodynamic stability has been linked to mutations associated with LCD that cause amyloid deposits. Mass spectrometry analyses of microdissected amyloid deposits from three different TGFBIp genotypes (A546D, A546D/P551Q, and V624M) of LCD showed elevated levels of peptides derived from the Tyr-571–Arg-588 amyloidogenic polypeptide region of FAS1-4. In addition, an increase in proteolytic susceptibility was observed in a region including residues Phe-515–Arg-533, located in the N-terminal of FAS1-4, compared with normal corneal TGFBIp (12–14). Deposits derived from GCD cases (13, 15) do not share the LCD characteristics, suggesting that distinct mechanisms are involved in these two types of corneal dystrophies. Intriguingly, protein deposits from all analyzed cases of LCD show accumulation of the serine protease high-temperature requirement A1 (HtrA1, UniProt Q92743, and MEROPS S01.277), possibly implicating this protease in the development of amyloid deposition in LCD (12). In contrast, HtrA1 is absent in the amorphous nonamyloid aggregates in GCD (13, 15). HtrA1 is a 51-kDa nonglycosylated serine protease that exists as a trimeric catalytic unit. It contains an N-terminal IGFBP-like domain, and a Kazal-like domain that can be removed by redox-mediated autolysis (16) and a C-terminal PDZ domain, that potentially may be involved in its association with fibrillar deposits (17). HtrA1 is expressed in the healthy cornea (1), and although its activation mechanism (18) and exact role are unclear, the homology to its bacterial counterpart, DegP, suggests that HtrA1 is involved in protein quality control (19, 20). HtrA1 is associated with amyloid deposits of immunoglobulin light chain amyloidosis (21), drusen deposits in age-related macular degeneration (22), and Alzheimer's disease (23). Concerning Alzheimer's disease, HtrA1 likely plays a role in amyloid protein turnover because the protease can degrade both monomeric and aggregated versions of several amyloid proteins (23, 24) and leads to the disintegration of amyloid fibrils (17).

In this study, the role of HtrA1 in the pathophysiology of *TGFBI*-linked corneal dystrophy was investigated by examining the ability of HtrA1 to cleave WT TGFBIp and seven mutants as well as WT FAS1-4 and six mutants. The TGFBIp isoforms included the two major LCD and GCD phenotypes caused by mutations in either the FAS1-1 or the FAS1-4 domain of TGFBIp. Our data show that TGFBIp may be the victim of counterproductive proteolysis, as HtrA1 attempts to degrade misfolded protein and inadvertently causes amyloid formation. We furthermore propose a model on how corneal proteostasis and the dual aggregation propensity of TGFBIp can lead to either the amorphous aggregates seen in GCD or the amyloid deposits observed in LCD.

## Results

### HtrA1 cleaves full-length TGFBIp protein in vitro

**Figure 1.**
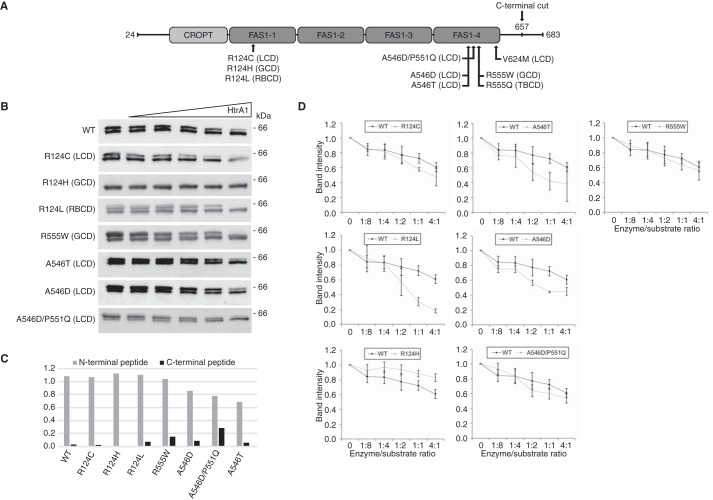
**Full-length TGFBIp is a substrate for HtrA1.** *A*, schematic representation of TGFBIp and its domain structure. Disease-linked mutations investigated in this study are marked along with the phenotype associated with the mutation. *B*, SDS-PAGE followed by immunoblotting of the HtrA1 titration series against WT and mutant full-length TGFBIp. *C*, SRM-LC-MS analysis of TGFBIp terminal peptides in the two bands observed by SDS-PAGE. The ratios (lower/upper band ratio) of the N-terminal and C-terminal peptides showed a close to 1:1 ratio for the N-terminal peptide, whereas the C-terminal peptide showed a low ratio across all genotypes, suggesting a C-terminal truncation in the lower band. *D*, densitometry of the HtrA1 titrations showed that the R124L mutation associated with RBCD and that mutations associated with LCD (R124C, A546T, A546D) increased *in vitro* TGFBIp turnover, whereas the LCD mutation A546D/P551Q showed a similar degradation trend as WT protein. The R555W mutation associated with GCD had no influence on the TGFBIp turnover rate, whereas the R124H mutation also classified as GCD resulted in increased proteolytic resistance against HtrA1. In general, HtrA1 showed a preference for full-length TGFBIp, causing a shift toward the C-terminally truncated TGFBIp variant before further processing. Experiments were performed in technical triplicate, and S.D. *error bars* are depicted for each titration point.

We expressed and purified seven mutant TGFBIp isoforms in addition to the WT protein (Fig. 1*A*). The seven TGFBIp mutants included the mutations R124C, R124H, and R124L, which are situated in the FAS1-1 domain of TGFBIp and are frequently observed *in vivo*, and the biologically relevant mutations A546D, A546D/P551Q, A546T, and R555W, which are located in the FAS1-4 domain of TGFBIp. Except for the R124L and A546T isoforms, we have previously characterized TGFBIp protein deposits of the remaining five mutant TGFBIp isoforms in corneal tissues (12–15, 25) of patients with LCD or GCD. This information allows us to compare the *in vitro* data presented herein to previously published *in vivo* data. The purified recombinant TGFBIp protein migrated as a double band when analyzed by SDS-PAGE (Fig. 1*B*) for all isoforms except for the R124H mutant. We used a single reaction monitoring MS assay (LC-SRM-MS) to delineate the nature of this double band. By monitoring a tryptic peptide at each terminal of TGFBIp, we identified the lower band as a C-terminally truncated TGFBIp isoform (Fig. 1*C*). The R124H mutant appears only as the C-terminally truncated protein.

Because HtrA1 accumulates in LCD TGFBIp deposits *in vivo*, we tested its activity toward the eight TGFBIp isoforms using a titration series of HtrA1 (Fig. 1). The eight TGFBIp isoforms were ranked from least to most susceptible to proteolysis as follows: R124H<WT<R555W<A546D/P551Q<R124C<A546D<A546T<R124L (Fig. 1*D*). The results revealed that the GCD mutants R124H and R555W were similarly or even more resistant to proteolysis than WT TGFBIp. The remaining mutant isoforms, all representing LCD except the R124L mutant, which causes Reis-Bücklers corneal dystrophy (RBCD), were more susceptible to proteolysis. Of note, HtrA1 showed a preference for full-length TGFBIp over the C-terminal truncated isoform and appeared to cause a shift from the upper band to the lower band over time (Fig. 1*B*). Interestingly, the R124H mutant appeared as only one band and thus seemed resistant to HtrA1 proteolysis. Whether the apparent proteolytic resistance is a consequence of the R124H mutation altering the structure or binding of HtrA1 to the C-terminal is required for the further proteolysis of TGFBIp is unknown. Together, these data support the idea that HtrA1 is involved in the natural turnover of TGFBIp in the healthy cornea as WT recombinant protein is processed and that introducing a mutation into TGFBIp affects its turnover rate.

### FAS1-4 mutations associated with LCD lead to HtrA1-mediated degradation

**Figure 2.**
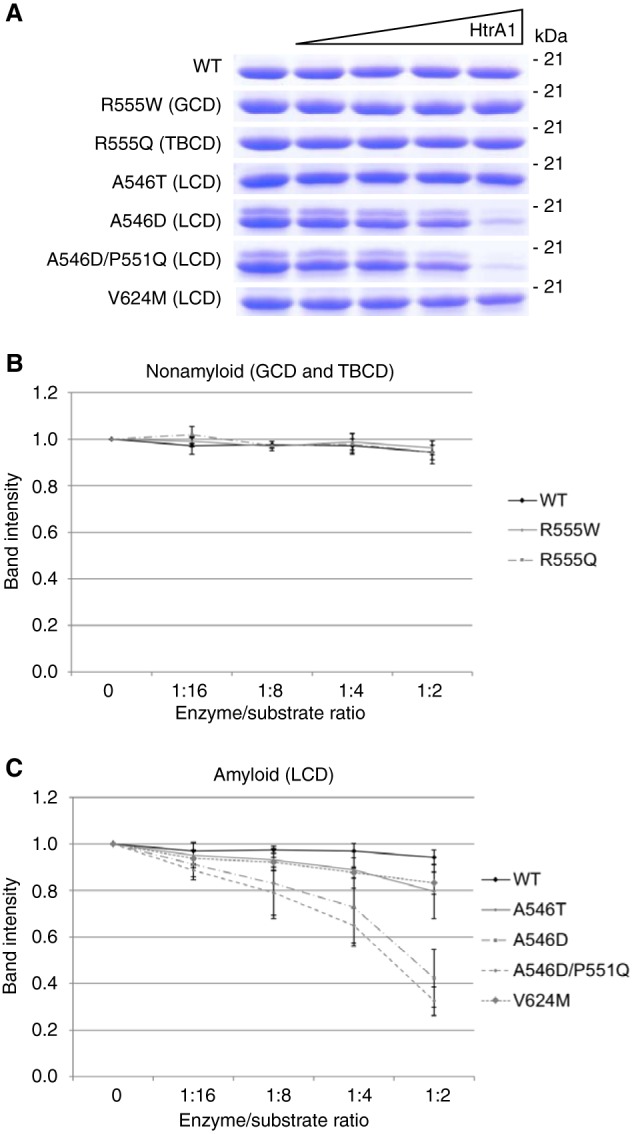
**Mutation in FAS1-4 dictates the turnover rate.** *A*, SDS-PAGE of the HtrA1 titration series against the WT and mutant FAS1-4 domains of TGFBIp. *B* and *C*, densitometric analyses showed (*B*) proteolytic resistance against HtrA1 by mutants associated with the nonamyloidogenic phenotypes GCD (R555W) and Thiel-Behnke corneal dystrophy (*TBCD*) (R555Q); (*C*) proteolytic sensitivity of mutations giving rise to LCD. WT FAS1-4 was minimally processed by HtrA1. Experiments were performed in technical triplicate, and S.D. *error bars* are depicted for each titration point.

The normal metabolism of corneal TGFBIp occurs by N-terminal trimming and produces fragments containing the C-terminal FAS1-4 domain. Therefore, we tested the ability of HtrA1 to cleave the WT FAS1-4 domain and six FAS1-4 mutants (Fig. 2). The double bands seen for the A546D and A546D/P551Q FAS1-4 domains (Fig. 2*A*) are, as shown previously (26), because of deamidations. The deamidations did not affect HtrA1 specificity and activity because both bands were degraded to the same extent. HtrA1 titration against the six mutant FAS1-4 domains separated the mutants into two distinct groups. One group was resistant to HtrA1 proteolysis and contained WT and the R555W and R555Q mutations associated with the nonamyloidogenic phenotypes GCD and Thiel-Behnke corneal dystrophy (TBCD) (Fig. 2*B*). The other group was susceptible to HtrA1 proteolysis and contained the A546T, A546D, A546D/P551Q, and V624M mutations, all representing the amyloidogenic phenotype LCD (Fig. 2*C*). In particular, the two A546D-containing mutants were markedly more susceptible to proteolysis than A546T and V624M, which showed similar sensitivity to proteolysis. The data suggest that the FAS1-4 domain is not a substrate for HtrA1 in healthy individuals. However, the destabilizing mutations associated with LCD change TGFBIp into a substrate for HtrA1.

### HtrA1 cleaves near the amyloidogenic region of FAS1-4 in TGFBIp

**Figure 3.**
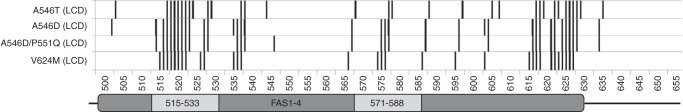
**FAS1-4 mutations cause alternative HtrA1 degradation.** The introduction of an *in vivo* relevant mutation in the FAS1 domain of TGFBIp influenced its degradation by HtrA1. The major cleavage sites, represented by more than eight spectra in the LC-MS/MS analysis, became more abundant in mutant proteins associated with the LCD phenotype (A546T, A546D, A546D/P551Q, and V624M). Cleavage sites appeared in and near the amyloidogenic 571–588 region in FAS1-4. Samples were analyzed in technical triplicate.

We analyzed the HtrA1 digest of FAS1-4 mutants associated with LCD (A546T, A546D, A546D/P551Q, and V624M) by MS to map the HtrA1 cleavage sites (P1 residue of both the N and C termini) across the various FAS1-4 mutants (Fig. 3). The mutants underwent extensive cleavage in two main clusters located in the peptide regions Phe-515–Ala-525 and Glu-615–Pro-635. In addition, all amyloidogenic mutants underwent additional peptide bond cleavage in the region between the two main proteolytic clusters. These additional cleavage sites lie near or within the amyloidogenic region of TGFBIp (residues Tyr-571–Arg-588) located in FAS1-4, and we suggest that HtrA1 contributes to the development of LCD by generating amyloidogenic peptides.

### HtrA1 proteolysis of LCD-linked FAS1-4 leads to aggregation

**Figure 4.**
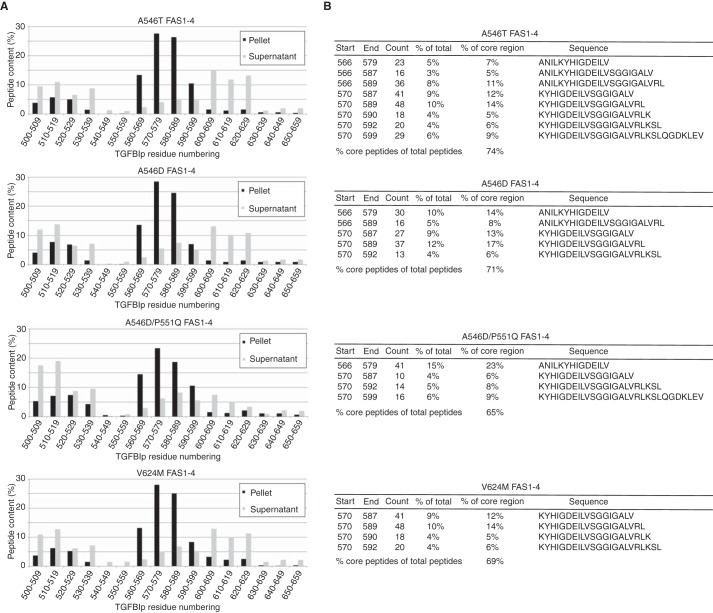
**HtrA1-generated FAS1-4 fibrils correlate with *in vivo* findings.** *A*, the LCD-linked mutant FAS1-4 was incubated with HtrA1, and the degradation products were able to form fibrils. The LC-MS/MS analysis of the pellet and supernatant showed the accumulation of peptides spanning the *in vivo* observed amyloidogenic region (Arg-571–Tyr-588) situated in FAS1-4 of TGFBIp. *B*, peptides spanning the amyloidogenic region constituted between 65 and 74% of the fibril matter, mostly represented by four to eight polypeptides. *Start* and *End* define the first and last residue number of the observed peptide. *Count* displays the total number of observed peptides. % *of total* represents the percentage of a peptide with respect to all identified peptides, whereas % *of core region* represents the percentage of a peptide with respect only to peptides falling within the amyloidogenic region of FAS1-4. *Sequence* displays the amino acid sequence of the observed peptide.

We addressed the aggregation propensity of HtrA1-generated FAS1-4 peptides by incubating monomeric FAS1-4 mutants (A546T, A546D, A546D/P551Q, and V624M) with HtrA1 for 21 h. After an additional 4 days of sample incubation, the pellets were separated from the supernatant by centrifugation. Both fractions were analyzed by LC-MS/MS, and the identified peptides were binned in 10–amino acid residue intervals across the FAS1-4 domain. A similar pattern was apparent for all amyloidogenic mutant FAS1-4 isoforms (Fig. 4*A*). The pellet fractions mainly consisted of peptides spanning the Gly-560–Leu-589 region of TGFBIp and, to a lesser extent, the Pro-500–Glu-529 region. The supernatant, on the other hand, showed the highest peptide counts outside the Gly-560–Leu-589 region (Fig. 4*A*). Hence, HtrA1 was able to generate TGFBIp peptides with the propensity to form insoluble aggregates consisting of peptides derived from the amyloidogenic region of FAS1-4 in TGFBIp. The number of peptides falling within the amyloidogenic region of TGFBIp constituted between 65 and 74% of all peptides identified in the pellet fraction (Fig. 4*B*). The highest represented peptide in the core region varied among genotypes; however, they all shared the sequence 570-KYHIGDEILV-579 (Fig. 4*B*). Therefore, the peptide region Lys-570–Val-579 appears to be essential for the progression of LCD if the disease is caused by mutations in the FAS1-4 domain.

### HtrA1-driven aggregates are amyloid in nature

**Figure 5.**
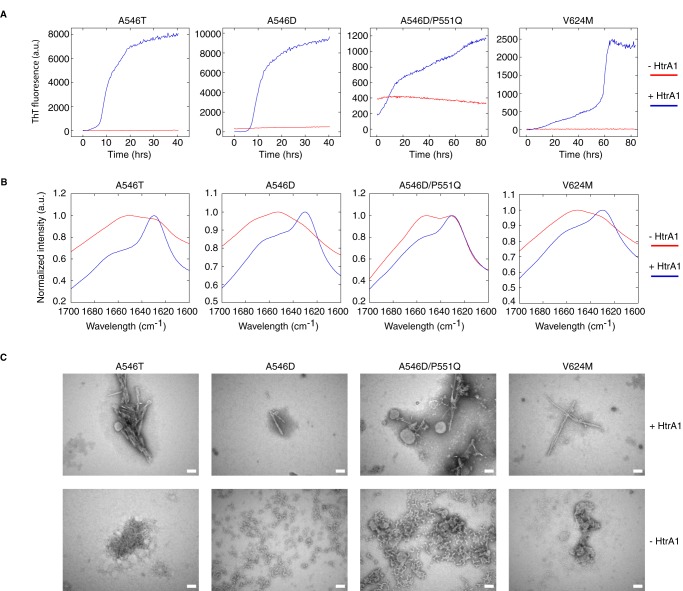
**HtrA1-generated fibrils are amyloid in nature.** *A*, thioflavin T fluorescence as a function of time for 24 μm LCD-linked FAS1-4 mutants preincubated with and without 2 μm HtrA1. A546D and A546D/P551Q both show some ThT fluorescence at the beginning of the assay, indicating nonnative structures for these two mutants. Graphs are representatives of technical triplicates performed for each mutant. *B*, FTIR spectroscopy showing the distinct amyloid peak at 1627–1630 cm^−1^ for all four samples preincubated with HtrA1. Graphs are averages of 64 technical measurements performed on each mutant. *C*, TEM micrographs of samples showing linear fibers for samples incubated with HtrA1. HtrA1-deficient A546T and V624M samples show small nonamyloid aggregates, whereas A546D and A546D/P551Q samples show larger nonamyloid aggregates, which likely play a role in the ThT signal observed for these mutants. The *scale bar* is 100 nm. *a.u.*, arbitary units.

HtrA1-generated fibrils were verified to be amyloid structures based on thioflavin T (ThT) fluorescence, FTIR, and transmission EM (TEM) (Fig. 5). FAS1-4 mutants were digested for 21 h with HtrA1, followed by 4 days of incubation. Increased ThT fluorescence as a function of time was observed for all four LCD-linked FAS1-4 variants in the presence of HtrA1, whereas ThT fluorescence was considerably reduced for the FAS1-4 variant alone, where only the A546D and A546D/P551Q mutants showed a slight fluorescence signal (Fig. 5*A*). The samples were further analyzed by FTIR spectroscopy, which showed a shift for the undigested FAS1-4 mutants from 1651–1653 cm^−1^ to 1627–1630 cm^−1^, values characteristic of β-sheet and β-amyloid-sheet, respectively (27), upon incubation with HtrA1 (Fig. 5*B*). The undigested A546D/P551Q mutant displayed both peaks, which could explain the slight ThT fluorescence signal observed for this mutant. Only the amyloid peak was observed for the digested A546D/P551Q mutant. Finally, the TEM micrographs showed elongated fibrils for FAS1-4 samples incubated with HtrA1, whereas nonfibrillar aggregates were observed for nonprotease-treated FAS1-4 mutants. The A546D and A546D/P551Q samples contained larger nonlinear aggregates than did A546T and V624M (Fig. 5*C*), which most likely accounted for the amyloid signatures observed in the ThT and FTIR experiments.

### HtrA1 is expressed throughout the healthy human cornea

**Figure 6.**
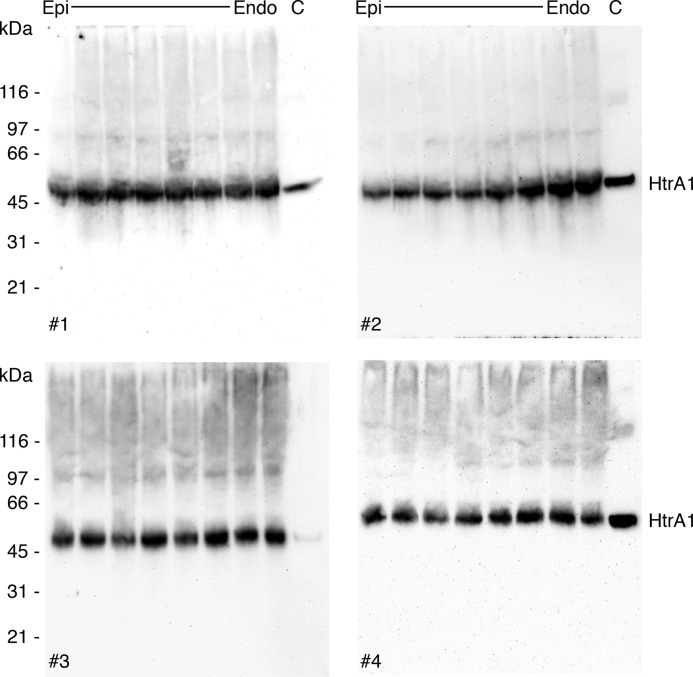
**HtrA1 is naturally present throughout the corneal stroma.** Human corneal sections were cut from the epithelium (*Epi*) toward the endothelium (*Endo*) of 8-mm diameter corneal buttons (biological replicates, *n* = 4). Tissue sections were subjected to SDS-PAGE followed by immunoblotting against HtrA1. HtrA1 was more or less uniformly distributed throughout the corneal stroma and, hence, not recruited only upon amyloid formation. This result implies that TGFBIp degradation products containing LCD-linked mutations in the FAS1-4 domain can be turned over immediately once they are formed.

To address whether HtrA1 is ubiquitously expressed throughout the stroma in healthy human corneas or accumulates only in amyloid deposits in patients' tissues, human corneal buttons were cut into 50 μm sections from the epithelium toward the endothelium. Approximately every second tissue section was subjected to SDS-PAGE and immunoblotting against HtrA1 (Fig. 6). The blots showed HtrA1 to be present throughout the corneal stroma, suggesting that HtrA1 is involved in normal corneal proteostasis and not induced only by LCD development.

### LCD tissue contains fewer FAS1-4 degradation products

**Figure 7.**
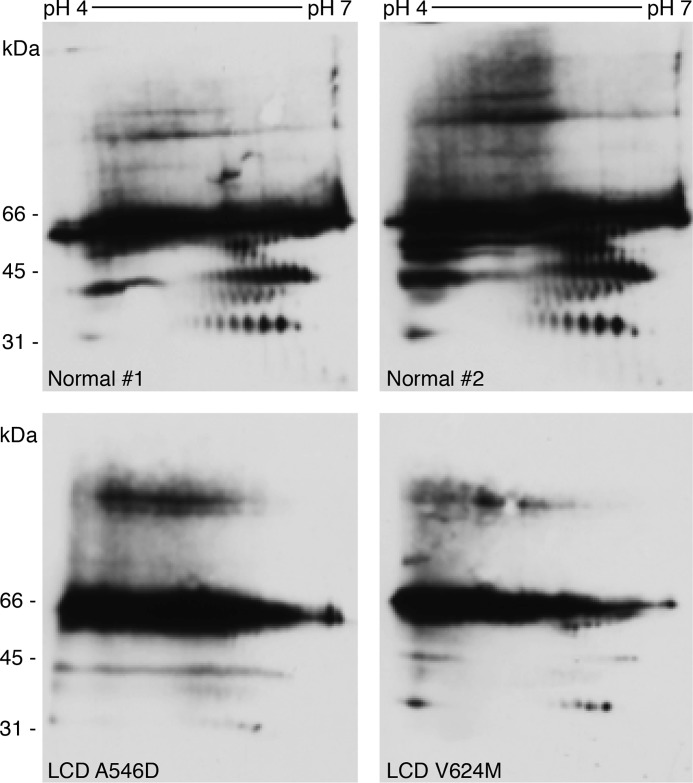
**FAS1-4 mutations lead to aberrant proteolytic processing of TGFBIp in the human cornea.** 2DE immunoblotting against TGFBIp in normal human tissue and LCD A546D and V624M corneal tissues is seen. Differential TGFBIp processing resulted in the detection of smaller amounts of degradation products by an antiserum recognizing the FAS1-4 domain of TGFBIp in LCD patient tissues. These data suggest an intensive turnover of mutated FAS1-4 in the cornea of LCD patients, possibly involving HtrA1 proteolysis. The pictures are representative of experimental replicates performed for each tissue. Normal 1 and 2 are biological replicates.

The *in vivo* processing of mutant TGFBIp was examined by visualizing the TGFBIp degradation patterns in patient tissue (A546D and V624M) and normal corneal tissue by 2D gel electrophoresis (2DE) and immunoblotting (Fig. 7). In the normal cornea, a characteristic degradation pattern of TGFBIp is observed as a consequence of N-terminal trimming; accordingly, all observed degradation products contain the FAS1-4 domain (2). Interestingly, A546D and V624M patient tissue had a reduced amount of degradation products, and the characteristic processing pattern was nearly undetectable. Notably, the antiserum recognizes only the FAS1-4 domain of TGFBIp; therefore, the observed TGFBIp degradation products, particularly in normal tissue, contained the FAS1-4 domain. As the FAS1-4 domain becomes a substrate for HtrA1 upon the introduction of a mutation associated with LCD (Fig. 2), the 2DE immunoblot data implied that TGFBIp degradation products spanning the FAS1-4 domain are consumed more rapidly by HtrA1, thus generating amyloidogenic core peptides situated in the FAS1-4 domain.

## Discussion

### TGFBIp is a substrate for HtrA1

Several extracellular proteins have been characterized as *in vitro* or *in vivo* substrates for HtrA1, such as fibronectin, fibromodulin, biglycan, aggrecan, and fibulin-5 (28), and our *in vitro* data add TGFBIp to this list of substrates. We showed that HtrA1 was capable of cleaving WT TGFBIp to a minor extent, and, except for R124H, all remaining TGFBIp mutants were processed to the same or a higher degree than WT protein, with the highest preferences for mutant isoforms associated with the RBCD and LCD phenotypes (Fig. 1). Previous results from our group have shown extensive corneal TGFBIp processing in healthy tissue, of which the smallest degradation product was FAS1-4 (2). As a model for the FAS1-4-containing degradation product and to assess its potential role as an HtrA1 substrate, we recombinantly expressed the FAS1-4 domain and subjected it to HtrA1-mediated degradation. Proteolysis of the WT and mutant FAS1-4 domains indeed showed that HtrA1 preferentially degraded FAS1-4 carrying a mutation associated with the LCD phenotype (Fig. 2). In contrast, WT FAS1-4 was a poor substrate for HtrA1 (Fig. 2), and the physiological involvement of HtrA1 in processing major TGFBIp degradation products containing the FAS1-4 domain is likely negligible. Collectively, our *in vitro* data support a role for HtrA1 in the turnover of disease-associated full-length TGFBIp. Mutations change FAS1-4 into suitable substrates for HtrA1 and thereby enable the aberrant processing of TGFBIp, observed in corneal tissue from LCD patients (Fig. 7).

### TGFBIp and HtrA1 are both expressed throughout the corneal stroma

We have previously shown that the major fraction of corneal mouse TGFBIp originated from the corneal epithelium (6). Furthermore, quantification showed abundant HtrA1 protein in the corneal proteome of the endothelial layer, including Descemet's membrane (1). We speculate that TGFBIp migrates from the epithelium toward the endothelium, where it encounters HtrA1 secreted from the endothelium. Our data show that HtrA1 is expressed throughout the stroma of normal corneas (Fig. 6). This information suggests the possibility that LCD-linked mutant TGFBIp is immediately exposed to HtrA1-mediated degradation once secreted by the epithelium. For disease-associated TGFBIp containing FAS1-4 mutations, this would lead to an increased turnover of the FAS1-4 species and the generation of amyloidogenic core peptides prone to aggregate formation. Interestingly, there appears to be a correlation between specific mutations leading to LCD, time of onset before deposit manifestations are observed, and how deeply amyloid deposits extend into the corneal stroma, from the epithelium toward the endothelium (29). This extension may directly correlate with the severity of the destabilizing mutation in the FAS1-4 domain of TGFBIp.

### HtrA1 degradation product may inadvertently fuel the progression of LCD

We have previously shown that the synthetic Tyr-571–Arg-588 amyloidogenic core peptide can form fibrils (30), leaving the possibility that digested FAS1-4 liberates the amyloidogenic core region, allowing fibril formation. In our *in vitro* experiments, HtrA1 was capable of digesting monomeric LCD-linked mutant FAS1-4, leading to amyloid fibril formation by peptides containing the amyloidogenic region (Figs. 3–5). Consequently, HtrA1 may play an undesirable key role in the progression of amyloid deposits in LCD caused by a mutation in FAS1-4 and therefore may be a potential target for LCD treatment. We cannot conclude whether the amyloidogenic region reported for FAS1-1, Leu-110–Glu-131 (31), accumulates upon HtrA1 proteolysis, as we were unable to express and purify the FAS1-1 domain. HtrA1 accumulates in amyloid deposits caused by both FAS1-1 and FAS1-4 mutations; however, FAS1-1 deposits did not show the same pronounced accumulation of the Tyr-571–Arg-588 amyloidogenic core region observed in deposits of FAS1-4 mutations (12–15). These results suggest that other amyloidogenic core regions in TGFBIp may constitute the amyloid deposits of FAS1-1–linked LCD. Altogether, our data support that HtrA1 unintentionally supplies building blocks to the amyloid fibril in its attempt to remove misfolded and mutated TGFBIp.

### Changes in TGFBIp turnover lead to either GCD or LCD

**Figure 8.**
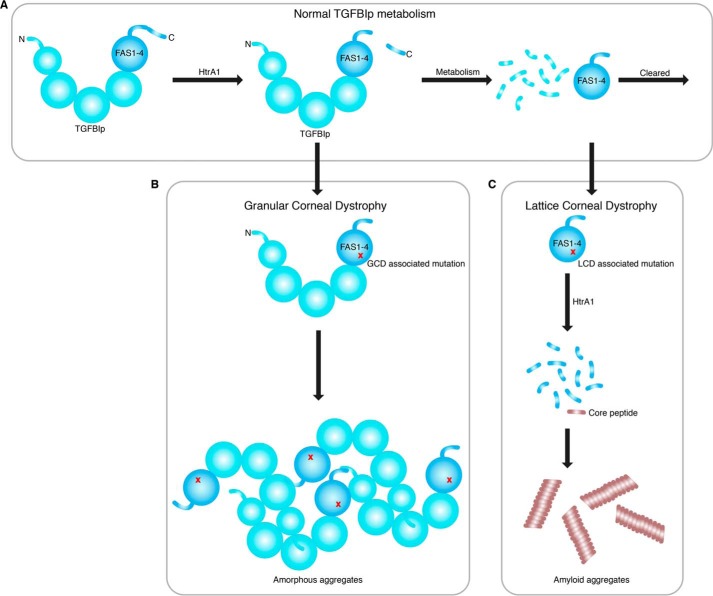
**Proposed mechanisms involved in *TGFBI*-linked corneal dystrophies.** *A*, normal corneal TGFBIp turnover where TGFBIp is first cleaved in its C-terminal, possibly by HtrA1. Then, it undergoes extensive N-terminal processing by the proteolytic machinery in the cornea, leading to FAS1-4–containing degradation products, which are cleared by an unknown mechanism. *B*, in GCD, the structural and proteolytic stability of TGFBIp increases, resulting in a slower turnover of the protein. This slower turnover leads to a buildup of TGFBIp, which eventually forms amorphous aggregates upon reaching a critical concentration. *C*, in the case of LCD, the TGFBIp structure is compromised by a mutation that increases its turnover or leads to alternative degradation pathways. LCD caused by FAS1-4 mutations allows the serine protease HtrA1 to turn over this domain, thereby liberating the amyloidogenic core region of TGFBIp. Upon reaching a critical concentration, the TGFBIp core peptide starts to form amyloid aggregates.

We propose that the mutation-induced changes in the normal corneal TGFBIp turnover (Fig. 8*A*) govern the phenotypic outcome (Fig. 8). Mutations associated with GCD lead to an increase in structural and proteolytic stability and an increased resistance to degradation by the proteolytic machinery in the cornea. This resistance causes a build-up of mutant TGFBIp in the cornea, eventually reaching a critical concentration and driving the protein into insoluble aggregates (Fig. 8*B*). In contrast, mutations associated with LCD destabilize the structure and increase TGFBIp turnover. In the case of FAS1-4 mutations, this domain becomes a substrate of HtrA1 and hence introduces an alternative turnover pathway (Fig. 8*C*). Whether FAS1-1 and FAS1-4 mutations share the same mechanism of protein aggregation is unclear. If so, one would expect all mutations in FAS1-1 and FAS1-4 that cause the same phenotype to be situated in close spatial proximity within the protein structure, thereby affecting the same local milieu of the protein. However, this pattern is not observed in the TGFBIp crystal structure (8). The disease-causing mutational hot-spot Arg-124 faces away from the FAS1-4 domain, and the many FAS1-4 mutations face away from Arg-124. Therefore, it is likely that different mechanisms take place whether the mutation is situated in FAS1-1 or FAS1-4, but both result in the same phenotype. This interpretation is supported by our *in vivo* analyses of mutated FAS1-1 and FAS1-4 deposits in LCD patient tissues (12–15). Mutation in FAS1-4 caused the accumulation of a specific FAS1-4 polypeptide region, which was absent in the R124C FAS1-1–linked LCD deposits. However, HtrA1 was associated with both the FAS1-1– and FAS1-4–related deposits, suggesting common features and implying that a general treatment directed at HtrA1 may be possible. Interestingly, others have shown that a 22–amino acid residue region spanning residue Arg-124 in FAS1-1 is capable of forming amyloid material *in vitro* (31) and most likely represents the amyloidogenic core region in FAS1-1–linked LCD patients. This possibility is further supported by the observation of *in vivo* accumulation of N-terminal degradation products of corneal TGFBIp in R124C patient tissue (32). Of note, the suggested FAS1-1 amyloidogenic core region becomes susceptible to fibrillation upon the introduction of a mutation, whereas the amyloidogenic region in FAS1-4 does not need any mutation to form amyloid material. Therefore, a change in the extracellular environment and not a mutation may be sufficient to cause TGFBIp aggregation, as observed in Fuchs endothelial corneal dystrophy. Here, TGFBIp is up-regulated and shown to be a part of the protein aggregates observed in guttae formation in Fuchs endothelial corneal dystrophy patients (33, 34).

## Conclusion

Several studies point toward aberrant proteolytic processing of TGFBIp as a crucial step toward the development of corneal dystrophy, either by causing a shift in the normal TGFBIp turnover rate or by abnormal corneal TGFBIp degradation (12–15, 25, 32). Thus, targeting the proteolytic machinery involved in TGFBIp processing may provide a way to prevent or delay the disease progression of *TGFBI*-linked corneal dystrophy. The serine protease HtrA1 has previously been associated with *in vivo* TGFBIp deposits in LCD. In this study, we show that TGFBIp is a substrate of HtrA1 and that mutations in TGFBIp allow aberrant processing by HtrA1 that may inadvertently promote amyloidogenesis in LCD.

## Experimental procedures

### Corneal tissue

Human postmortem corneal tissue (*n* = 5; average age, 82 years old) and corneal LCD patient tissue that had been studied previously (12, 13) were used with permission from the Regional Committee for Scientific Medical Ethics, Denmark, and according to the Declaration of Helsinki.

### Recombinant FAS1-4 domain expression and purification

FAS1-4 WT and mutated variants (residues 502–657 of TGFBIp, Swiss-Prot accession number Q15582, with two additional amino acids Ala-Gly at the N terminus) were expressed as N-terminally His-tagged SUMO-FAS1-4 fusion proteins in the chemically competent *Escherichia coli* strain One Shot® BL21(DE3) (Invitrogen) grown in lysogeny broth medium (Sigma-Aldrich) containing 50 μg/ml kanamycin (Sigma-Aldrich). Expression of FAS1-4 was induced with 1 mm isopropyl β-d-1-thiogalactopyranoside (IPTG) (PanReac AppliChem) at an absorbance of 0.4–0.7 at 600 nm. Three h after induction, cells were harvested by centrifugation at 6000 × *g* for 15 min and stored at −80 °C until further use.

The harvested cells were resuspended in 50 mm Tris-HCl, 500 mm NaCl, pH 7.6, containing a cOmplete, EDTA-Free Protease Inhibitor Mixture Tablet (Roche Diagnostics) and sonicated for 3 × 8 min at 70% amplitude with 0.5-s cycles while kept on ice. Cell debris was removed by centrifugation for 15 min at 10,000 × *g*, followed by 15 min of centrifugation at 25,000 × *g*. The supernatant was filtered using a 0.22-μm filter and supplemented with 20 mm imidazole before being applied to a 5-ml HisTrap HP column (GE Healthcare) equilibrated in 50 mm Tris-HCl, 500 mm NaCl, 20 mm imidazole, pH 7.6. The protein was eluted using a stepwise gradient of 80 mm, 200 mm, 300 mm, 400 mm, and 500 mm imidazole, and protein fraction purity was assessed by SDS-PAGE (SDS-PAGE). The purified protein was dialyzed overnight at 4 °C into 50 mm Tris-HCl, 100 mm NaCl, pH 7.6, and the SUMO moiety was subsequently cleaved off by the addition of 5 mm DTT and 4 unit/ml SUMO Protease (Invitrogen) followed by incubation at room temperature for 4 h. FAS1-4 protein was dialyzed into PBS and repurified by nickel-chelating chromatography. Pure FAS1-4 protein was collected in the flow-through and stored at −80 °C.

### Recombinant full-length TGFBIp expression and purification

Full-length TGFBIp variants were expressed in the human cell line FreeStyle^TM^ 293-F (Invitrogen) after transfection using the polyethylenimine method. At 72 h after transfection, the culture supernatant was collected by centrifugation for 2 min at 1000 × *g* and incubated with 1 mm PMSF and 5 mm EDTA for 30 min before being dialyzed into buffer A (20 mm Tris-HCl, pH 7.5), followed by purification by affinity chromatography using a 5 ml HiTrap Heparin HP column (GE Healthcare). A linear gradient of buffer A to buffer B (20 mm Tris-HCl, 1 m NaCl, pH 7.5) was used to elute the protein. Selected fractions were analyzed by SDS-PAGE, and fractions containing TGFBIp were pooled and dialyzed into buffer A. Subsequently, TGFBIp was purified from the dialysate by anion-exchange chromatography using a 5 ml HiTrap Q HP column (GE Healthcare) and eluted with a linear gradient from buffer A to buffer B. Selected fractions were analyzed by SDS-PAGE, and fractions containing TGFBIp were pooled and dialyzed into PBS prior to storage at −80 °C.

### Recombinant expression and purification of HtrA1

HtrA1 cDNA inserted into the pCMV6-XL4 vector using NotI-NotI restriction sites was obtained from OriGene (NM_002775). The translational and secreted product resulted in the full-length HtrA1 serine protease (amino acid residues 23–480) without any purification tags at its termini. HtrA1 plasmid was transfected using Fectin 293 (Life Technologies) transfection reagent into Freestyle 293-F cells cultivated in Freestyle 293 Expression Medium (Life Technologies) at a cell density of 1 × 10^6^ cells/ml. After incubation for 48 h, the culture supernatant was collected and dialyzed overnight at 4 °C into 20 mm phosphate buffer, pH 7. The dialyzed medium was subjected to cation exchange chromatography (HiTrap SP HP, GE Healthcare) and developed using a 1 m NaCl gradient. The HtrA1 fraction was subsequently dialyzed into 20 mm phosphate buffer, pH 7, at 4 °C and subjected to heparin chromatography (HiTrap Heparin, GE Healthcare). The HtrA1 fraction was pooled and dialyzed into PBS, pH 7.4, and the purity, concentration, and activity toward β-casein were estimated and tested by SDS-PAGE. A mock transfection and purification was performed by transfecting cells without plasmid and by using LC fractions corresponding to fractions that contained HtrA1 in the HtrA1 purification. LC-MS/MS analysis to identify potential contaminating proteases was performed on the final material of the mock purification and did not identify any contaminating proteases (data not shown).

### HtrA1 proteolysis of soluble TGFBIp and FAS1-4

The activity of HtrA1 toward full-length TGFBIp variants was tested by performing an HtrA1 titration series against a fixed amount of full-length TGFBIp. To 0.25 μg of full-length TGFBIp was added 0, 31.5, 62.5, 125, 250, and 1000 ng of HtrA1, separately, and the mixtures were incubated overnight at 37 °C before being inhibited by 2 mm PMSF. A control with the highest amount of HtrA1 alone was included. Samples were subjected to SDS-PAGE followed by the detection and quantification of TGFBIp by immunoblotting using a rabbit polyclonal antibody against the TGFBIp peptide 571-YHIGDEILVSGGIGALVR-588 (EZBiolab) and a Cy3-labeled secondary antibody (Sigma-Aldrich). All samples were analyzed in triplicate. Comparison between the WT and mutant titration series was performed by normalizing each titration point to the sample without HtrA1 for a given titration series.

To test the activity of HtrA1 toward FAS1-4 variants, an HtrA1 titration series was performed against a fixed amount of FAS1-4. To 2 μg of FAS1-4 was added 0, 0.125, 0.25, 0.5, and 1 μg of HtrA1, separately, and incubated overnight at 37 °C before being inhibited by 2 mm PMSF. A control with the highest amount of HtrA1 alone was included. Samples were subjected to SDS-PAGE, and densitometry of the bands was performed on a FluorChem Q scanner using the AlphaView software (ProteinSimple). All samples were analyzed in triplicate and normalized to the sample without HtrA1.

Furthermore, an additional HtrA1 digest of LCD-associated FAS1-4 mutants using the highest protease concentration was included and subjected to LC-MS/MS analysis. HtrA1 cleavage sites observed more than eight times were compared between FAS1-4 variants.

### LC-MS/MS analysis

Samples for MS were analyzed by nanoflow LC-tandem MS (LC-MS/MS) using an Easy-nLC II system (Thermo Scientific) coupled directly to a TripleTOF 5600 mass spectrometer (AB Sciex) operated under Analyst TF 1.6.0 control. Samples were loaded on a 0.1 × 20 mm 3-μm C18 trap column and a 0.075 × 150 mm 3-μm C18 analytical column, both pulled and packed in-house. Peptides were eluted and electrosprayed directly into the mass spectrometer using a 20 min gradient from 5–40% acetonitrile in 0.1% acetic acid at a flow rate of 250 nl/min. Data were acquired using an ion spray voltage of 2.3 kV, a curtain gas of 30, and an interface heater temperature of 150 °C. For information-dependent acquisition, survey scans were acquired in 250 ms, and as many as 25 product ion scans were collected if exceeding a threshold of 150 counts per second and with a +2 to +5 charge-state. A sweep collision energy setting of 35 ± 15 eV was applied to all precursor ions for collision-induced dissociation. No dynamic exclusion was applied.

### MS database search

MS Raw data were converted to mgf format using AB SCIEX MS Data Converter beta 1.1 (AB Sciex) and the “proteinpilot MGF” parameters and searched using an in-house Mascot search engine (Matrix Science, London, UK; version: 2.3.02) against the Swiss-Prot database (version: 2012_05). No enzyme was selected, and missed cleavage was set to 0. No fixed modifications were added. Instead, the oxidation of methionine and proline were included as variable modifications. The MS tolerance and MS/MS tolerance were 10 ppm and 0.2 Da, respectively, and an ESI-QUAD-TOF system was set as the instrument used. All data sets were searched using a *p* value < 0.01, and peptides with an ion score lower than 30 were rejected. All searched data were imported to and processed in MS Data Miner (version 2.0) (35).

### Proteolysis of FAS1-4 in amyloid formation

The propensity for HtrA1-generated FAS1-4 peptides to form aggregates was investigated by incubating 24 μm (32 μg) of mutant FAS1-4 (A546D, A546D/P551Q, A546T, and V624M) with or without 2 μm (8 μg) HtrA1 in PBS, pH 7.4, for 21 h at 37 °C. The FAS1-4 samples with and without prior HtrA1 digestion were subsequently incubated at 37 °C with double orbital shaking for 2 min at 500 rpm every 20 min. The fibrillation was carried out in triplicate in the presence of ThT to monitor fluorescence over time (described in detail below). All samples were divided into pellet and supernatant fractions by 40,000 × *g* centrifugation followed by FTIR, TEM, and LC-MS/MS. The identified TGFBIp peptides were divided into bins of 10–amino acid residues throughout the sequence, and each fraction was summed and plotted as an intensity (spectral count).

### ThT, FTIR, and TEM characterization

To verify the nature of the HtrA1-generated fibrils as amyloid, thioflavin T fluorescence was recorded as a function of time. FAS1-4 protein was incubated with and without the presence of HtrA1 using a final ThT concentration of 40 μm (Ex 450 nm, Em 480 nm). The samples were then further analyzed by FTIR spectroscopy on a Tensor 27 (Bruker) FTIR spectrophotometer equipped with a DTGS Mid-IR detector and a Golden Gate single-reflection diamond-attenuated total reflectance cell (Specac). Samples were placed on the ATR crystal and dried using dry nitrogen. Spectra were recorded from 4000–1000 cm^−1^ using a resolution of 2 cm^−1^ and 64 accumulations. The data were corrected for background and atmospheric interference and normalized to the peak intensity. Finally, TEM micrographs were taken for all samples. Five μl of sample were mounted on 400 mesh carbon-coated, glow-discharged nickel grids for 1 min, stained with 1 drop of phosphotungstic acid (PTA), pH = 7.0 and blotted dry on filter paper. EM was performed on a JEOL-1010 electron microscope (JEOL, Tokyo, Japan) operated at 60 keV. The microscope was coupled to an electron-sensitive CDC camera (KeenView, Olympus Soft Imaging Solutions GmbH, Münster, Germany). A grid-size replica (2160 lines/mm) was used for size determination.

### 2DE of human corneal tissue

Human LCD and WT corneal tissue samples were lyophilized for 6 h before homogenization in liquid nitrogen to generate corneal powder. The corneal powder was then subjected to 2DE lysis buffer followed by 2DE using a pH 4–7 strip and 10% gels and was immunoblotted. Rabbit antisera recognizing TGFBIp were used as the primary antibody at a 1:5000 dilution in 2% skimmed milk/TBS-T, and goat anti-rabbit HRP-conjugated antibody was used as the secondary antibody at a 1:10,000 dilution in 2% skimmed milk.

### Stromal HtrA1 distribution

Eight mm central postmortem human corneal buttons from three individuals were cut into sections of 50 μm from the epithelium toward the endothelium. Nine sections throughout the corneal stroma from each cornea were boiled in SDS sample buffer, leaving out the two outermost corneal cell layers. The samples were subjected to SDS-PAGE, followed by immunoblotting using HtrA1 antiserum. Blots were developed using the FluorChem Q scanner and the AlphaView software (ProteinSimple).

### LC-SRM analysis of TGFBIp terminal truncations

All but one of the expressed and purified TGFBIp isoforms resulted in two bands when analyzed by SDS-PAGE, most likely as a consequence of terminal truncation. To clarify at which terminal truncation occurs, a target SRM-MS–based analysis was performed to map tryptic peptides along the whole TGFBIp protein. The SRM assay was optimized using Skyline v.3.1.0 (36), in which 25 peptides with four transitions each were monitored. Stable isotope-labeled synthetic peptides (SpikeTides, JPT Peptide Technologies, Berlin, Germany) were used for assay development and as spike-in standard peptides in the in-gel digested TGFBIp samples. LC-SRM was performed using an EASY-nano LC (Thermo Scientific, Waltham, MA) connected in-line with a QTRAP 6500 (AB Sciex, Framingham, MA). Peptide samples were injected and trapped on a C18 precolumn (5 μm, 2 cm × 100 μm I.D., packed in-house), followed by elution to and separation on a 15-cm analytical column (75 μm I.D., packed in-house with RP ReproSil-Pur C18-AQ 3 μm resin (Dr. Maisch GmbH, Ammerbuch-Entringen, Germany) using a flow rate of 250 nl/min and a 20-min 5–35% B gradient (A solvent; 0.1% formic acid, B solvent; 0.1% formic acid and 100% acetonitrile). The SRM assay was run as a scheduled method using a 3-min window. The raw SRM data were imported to Skyline to obtain the L/H ratio of the tryptic TGFBIp peptides and subsequently exported to Excel. The summed intensities of four internal peptides of TGFBIp (77-STVISYECCPGYEK-90, 173-YHMVGR-178, 180-VLTDELK-186, and 220-ADHHATNGVVHLIDK-234) were used to normalize the upper and lower band intensities before determining the ratios of the N-terminal (28-SPYQLVLQHSR-38) and C-terminal (670-LAPVYQK-676) peptides between the lower and upper band. The R124H full-length TGFBIp mutant migrated as one band and was normalized to the WT full-length TGFBIp upper band.

## Author contributions

E. T. P., N. S. N., H. V., and J. J. E. conceptualization; E. T. P., N. S. N., and C. S. data curation; E. T. P., N. S. N., C. S., E. H. M., M. W. R., K. R., M. V. L., C. B. R., G. C., and M. R. formal analysis; E. T. P., H. V., and J. J. E. supervision; E. T. P., H. V., and J. J. E. funding acquisition; E. T. P. and N. S. N. methodology; E. T. P., N. S. N., and C. S. writing-original draft; J. J. E. resources; J. J. E. project administration; J. J. E. writing-review and editing.

## Footnotes

3 The abbreviations used are:

LCD: lattice corneal dystrophy
GCD: granular corneal dystrophy
SRM: single reaction monitoring
RBCD: Reis-Bücklers corneal dystrophy
ThT: thioflavin T
TEM: transmission EM
2DE: 2D gel electrophoresis
I.D.: inner diameter.
